# Long working hours and preventive oral health behaviors: a nationwide study in Korea (2007–2021)

**DOI:** 10.1265/ehpm.24-00102

**Published:** 2024-09-12

**Authors:** Seong-Uk Baek, Jin-Ha Yoon, Yu-Min Lee, Jong-Uk Won

**Affiliations:** 1 Department of Occupational and Environmental Medicine, Severance Hospital, Yonsei University College of Medicine, Seoul, Korea; 2 The Institute for Occupational Health, Yonsei University College of Medicine, Seoul, Korea; 3 Graduate School, Yonsei University College of Medicine, Seoul, Korea; 4 Department of Preventive Medicine, Yonsei University College of Medicine, Seoul, Korea

**Keywords:** Dental public health, Health behaviors, Health-related behaviors, Lifestyles, Oral hygiene, Overwork, Working time

## Abstract

**Background:**

This study explored the association between working hours and preventive oral health behaviors.

**Methods:**

In total, 48,599 workers (22,992 females) were included from the Korea National Health and Nutrition Examination Survey (2007–2021). Weekly working hours were self-reported. The following three preventive oral health behaviors were set as outcomes: participation in annual dental check-ups; adherence to the recommended toothbrushing frequency (≥twice a day); and use of interdental cleaning devices. We estimated odds ratios (ORs) and 95% confidence intervals (CIs) using logistic regression.

**Results:**

In male workers, the adjusted OR (95% CI) of the association between working ≥55 h/week and each outcome was 0.84 (0.77–0.92) for dental check-ups, 0.82 (0.72–0.94) for toothbrushing ≥twice a day, and 0.83 (0.76–0.92) for utilization of interdental cleaning device when compared to 35–40 h/week. In female workers, the adjusted OR (95% CI) of the association between working ≥55 h/week and each outcome was 0.79 (0.70–0.89) for dental check-ups, 0.88 (0.70–1.11) for toothbrushing ≥twice a day, and 0.80 (0.71–0.90) for utilization of interdental cleaning device when compared to 35–40 h/week. Additionally, low socio-economic status, such as low educational attainment, low income level, and blue-collar occupations, were major risk factors associated with non-adherence to preventive oral health behaviors in both male and female workers.

**Conclusions:**

Our study suggests that individuals who work long hours are more likely to exhibit undesirable oral health behaviors.

**Supplementary information:**

The online version contains supplementary material available at https://doi.org/10.1265/ehpm.24-00102.

## Graphical Abstract

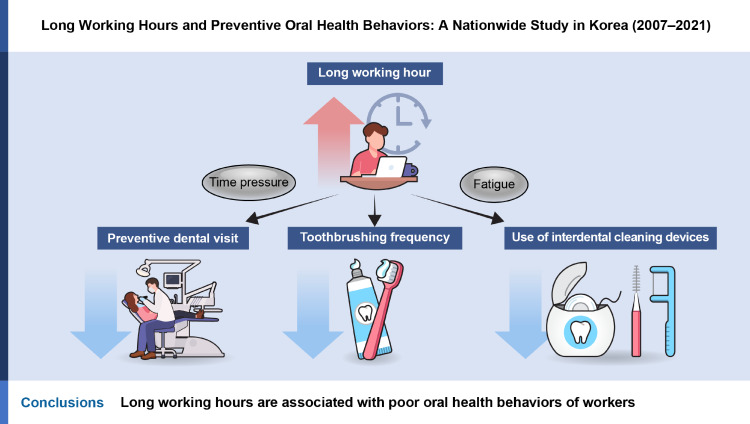

## 1. Background

Long working hours are a major global public health concern. A report conducted by the World Health Organization (WHO) and International Labour Organization (ILO) estimated that globally, approximately 488 million individuals engage in long working hours, defined as ≥55 h/week; in 2016, this caused over 745,000 overwork-related deaths [1]. A recent shift in the economic landscape, such as the emergence of the gig economy and Industry 4.0, has the potential to further expedite this trend [1, 2]. For decades, South Korea has been recognized as being among the countries with the longest annual working hours. This has heightened societal interest in the effects of long working hours [3, 4]. In South Korea, approximately 65% of the total population participates in economic activities [5], and approximately 16% of all workers work more than 55 h/week [6].

Recently, there has been increasing research interest in the potential impact of long working hours and overwork on the dental health of workers. Previous studies suggest that long working hours are associated with poor oral health conditions [7–9]. For instance, a previous study showed that working >52 h/week was associated with a higher likelihood of periodontitis among Korean workers [8]. Additionally, Japanese workers, who frequently work overtime, are more likely to experience untreated tooth decay or poor oral health-related quality of life [7, 9]. One potential mechanism through which long working hours may contribute to poor oral health is their negative impact on workers’ oral health behaviors [10]. For instance, previous studies suggest that long working hours may constrain time, preventing workers from scheduling preventive dental care visits or seeking dental care for symptom management [11–13].

Improving preventive oral health behaviors, such as maintaining good oral self-care habits and attending regular dental check-ups, is particularly important for the working population. It not only contributes to overall well-being but also has a substantial influence on work productivity [14, 15]. Engagement in regular dental check-ups, sufficient toothbrushing, and the use of interdental cleaning devices are essential factors that have been important indicators in the previous studies for understanding the epidemiology of oral health behaviors among Koreans [16, 17]. Recognizing the importance of preventive oral health behaviors, Korea’s health insurance covers one regular dental check-up per year. Although Korea has seen a gradual improvement in the quality of oral health behaviors among workers in recent decades, certain individuals in precarious positions, including non-standard workers, are more likely to exhibit poor oral health behaviors [18–20]. According to a recent study, approximately 45% of Koreans participate in regular dental check-ups, with lower participation rates observed in households with lower income levels [21]. Concerningly, the literature contains only a limited number of studies investigating the potential relationship between long working hours and preventive oral health behaviors. Some found that those working long hours were less likely to engage in dental check-ups [12, 13]. However, to our knowledge, the association between long working hours and adherence to recommended daily toothbrushing practices and the use of interdental cleaning devices has not been explored. Furthermore, the relationship between long working hours and oral health behaviors in the Korean population has not yet been investigated.

Therefore, this study investigated the relationship between long working hours and preventive oral health behaviors in the Korean population. Specifically, using a nationally representative sample of Korean workers, we aimed to verify the hypothesis that long working hours were negatively associated with dental check-up attendance, daily toothbrushing frequency, and the use of interdental cleaning devices in both male and female workers.

## 2. Materials and methods

### 2.1. Study sample

We used the Korea National Health and Nutrition Examination Survey (KNHANES), a population-based, repeated cross-sectional survey that includes a nationwide sample of the general Korean population [22]. The Korea Disease Control and Prevention Agency (KDCA) has conducted the KNHANES annually since 1998 to collect information on the health status of Koreans. The KNHANES employs a multi-stage clustering sampling method to select a nationally representative sample, in which enumeration districts in Korea were selected as the primary sampling unit and households in each region were selected as the secondary unit [23]. Face-to-face interviews were conducted by trained interviewers employed by the KDCA. For each survey year, the response rates ranged from 70% to 80% [23]. To mitigate bias arising from non-response and enhance the generalizability of the sample, sampling weights were assigned to survey participants [23].

**Fig. 1:**
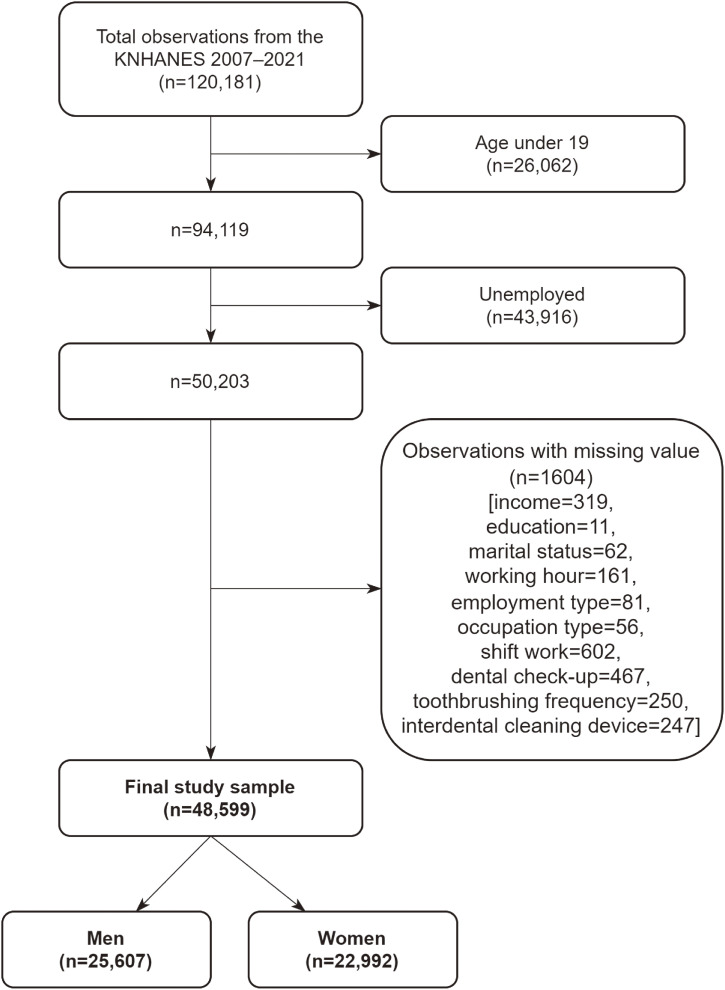
Flowchart of the selection process of the study sample

Since information on working hours has only been collected since 2007, we included KNHANES participants from 2007 to 2021. A flowchart of the study sample selection process is presented in Fig. 1. The inclusion criteria were as follows: individuals aged 19 years or older; workers (defined as those who engaged in economic activities), and observations without missing values. In total, 48,599 workers (25,607 males and 22,992 females) were included in the final sample. Approximately 3000–4000 individuals were included in each survey.

### 2.2. Data availability and ethics statement

The KNHANES dataset is available online at https://knhanes.kdca.go.kr [22]. The Institutional Review Board of Yonsei Health System reviewed and approved this study (IRB No. 4–2023–0959; approval date: September 13, 2023).

### 2.3. Variables

#### 2.3.1. Working hours

The main independent variable was the number of working hours per week. In each survey, participants were asked the following question: “What are the average working hours per week at your workplace? Taking into account overtime but not counting meal breaks” We categorized working hours per week as “<35 h,” “35–40 h,” “41–48 h,” “49–54 h,” and “≥55 h”, consistent with the categorization in previous WHO/ILO studies [24, 25]. Those working standard hours (35–40 h/week) were defined as the reference group.

#### 2.3.2. Preventive oral health behaviors

This study focused on three preventive oral health behaviors as follows: participation in dental check-ups; adherence to the recommended toothbrushing frequency (≥twice a day); and use of interdental cleaning devices. For the first outcome, survey participants were asked the following question: “Have you undergone a dental check-up within the past year to assess your oral health condition?”. Those who responded “yes” were categorized as having undergone a dental check-up. For the second outcome, survey participants were asked whether they had brushed their teeth at eight different time points during the previous day: before and after breakfast, before and after lunch, before and after dinner, after snacking, and immediately before sleeping. Thus, daily tooth brushing frequency ranged from 0–8. Those who brushed their teeth twice or more per day were categorized as adhering to the recommended daily toothbrushing frequency [26, 27]. For the third outcome, participants were asked whether they used dental floss or interdental brushes. Individuals who used one or both of these tools were classified as using interdental cleaning devices.

#### 2.3.3. Confounders

We selected the following covariates based on previous studies examining the association between long working hours and oral health [7–9]. Age was categorized as “19–29,” “30–39,” “40–49,” “50–59,” and “≥60.” Educational level was categorized as having completed “college or above”, “high school,” and “middle school or below”. Income level was categorized according to the quartile values of total household income in each survey year. Given the gradual inflation over the years, we categorized income levels into intervals for each year, resulting in varying cut-off values by year. The exact values for income level categorizations are provided in the analysis guidelines of the KNHANES [28]. Marital status was categorized as “married” and “unmarried or others (divorced, separated, widowed).” Employment status was categorized as “regular employment”, “fixed-term employment”, and “self-employment or other”. Occupation was categorized as “white collar”, “service or sales worker”, and “blue collar”, according to the Korean Standard Classification of Occupation. Managers, professionals and related workers; and clerks were categorized into “white collar workers.” Service workers and sale workers were categorized into “service or sale workers.” Skilled agricultural, forestry and fishery workers; craft and related trades workers; plant, machine operators and assemblers; and elementary workers were categorized into “blue collar workers.” Shift work was categorized as either “yes” or “no” based on whether the participant’s work schedule involved regular or irregular rotating shifts, or fixed evening or night working.

### 2.4. Statistical analysis

For the descriptive analysis, we examined the characteristics of study participants based on their weekly working hours. We then investigated the prevalence of each of the three preventive oral health behaviors according to weekly working hours.

We employed logistic regression analysis to explore the association between working hours per week and each oral health behavior. Both unadjusted and fully adjusted models were fitted to estimate the association between long working hours and each outcome. Subsequently, we investigated the association between long working hours and post-mealtime toothbrushing. Specifically, the association between working hours per week and toothbrushing after breakfast, lunch, and dinner were estimated. Effect sizes were presented as odds ratios (ORs) along with 95% confidence intervals (CIs). For additional analyses, we conducted stratified analyses to explored how the association between long working hours and preventive oral behaviors differs depending on occupational groups and employment types.

Statistical analyses were stratified by gender, considering that (i) there are gender differences in oral health behaviors [20, 29] and that (ii) previous studies report gender differences in the association between long working hours and oral health conditions [11, 13]. All data preparation, analyses, and visualizations were performed using R software (version 4.2.3; R Foundation for Statistical Computing, Vienna, Austria). Sampling weights were applied to account for the complex survey design of the KNHANES. The “survey” package was employed for the analysis of complex survey design [30]. The “svyglm” function was used for survey weight-adjusted logistic regressions.

## 3. Results

Table S1 presents the distribution of the study sample characteristics. Among male workers, 16.0% worked <35 h/week, 24.8% worked 35–40 h/week, 20.0% worked 41–48 h/week, 14.1% worked 49–54 h/week, and 25.1% worked ≥55 h/week. Among female workers, 35.0% worked <35 h/week, 26.7% worked 35–40 h/week, 16.1% worked 41–48 h/week, 8.0% worked 49–54 h/week, and 14.1% worked ≥55 h/week. In both genders, those who worked ≥55 h/week were predominantly older individuals, had lower educational attainment, had lower income levels, and had blue-collar occupations.

Figure S1 shows the weighted percentages of workers exhibiting each preventive oral health behavior. The percentage of workers exhibiting each oral health behavior was lower among individuals working ≥55 h/week compared with those working 35–40 h/week. Among men working ≥55 h/week, the percentages were 29.6% for dental check-ups, 85.4% for toothbrushing ≥twice a day, and 25.6% for use of interdental cleaning devices. Among women working ≥55 h/week, the percentages were 26.6% for dental check-ups, 91.4% for toothbrushing ≥twice a day, and 29.1% for use of interdental cleaning devices.

Table 1 shows the association between working hours per week and preventive oral health behaviors in men and women. In male workers, the adjusted OR (95% CI) of the association between working ≥55 h/week, when compared to 35–40 h/week, and each outcome was 0.84 (0.77–0.92) for dental check-ups, 0.82 (0.72–0.94) for toothbrushing ≥twice a day, and 0.83 (0.76–0.92) for utilization of interdental cleaning device. In female workers, the adjusted OR (95% CI) of the association between working ≥55 h/week, when compared to 35–40 h/week, and each outcome was 0.79 (0.70–0.89) for dental check-ups, 0.88 (0.70–1.11) for adherence to recommended toothbrushing frequency, and 0.80 (0.71–0.90) for utilization of interdental cleaning device. Additionally, workers with low socio-economic status, including those with low educational levels, low income levels, and blue-collar jobs, were less likely to engage in dental check-ups, brush their teeth twice or more per day, and use interdental cleaning devices, in both male and female workers. Table S2 shows that the negative association between long working hours and preventive oral health behaviors are observed across occupational groups and employment types.

**Table 1:** Association between working hours per week and each preventive oral health behavior.

	**Male**			**Female**		

	**Participation in dental check-up**	**Toothbrushing ≥twice a day**	**Use of interdental cleaning device**	**Participation in dental check-up**	**Toothbrushing ≥twice a day**	**Use of interdental cleaning device**

	**OR (95% CI)**	**OR (95% CI)**	**OR (95% CI)**	**OR (95% CI)**	**OR (95% CI)**	**OR (95% CI)**
**Participation in dental check-up**						
Working hours per week						
<35 h	1.02 (0.92–1.13)	0.80 (0.69–0.92)	1.02 (0.91–1.14)	1.01 (0.92–1.11)	1.01 (0.83–1.22)	1.20 (1.09–1.32)
35–40 h	Reference	Reference	Reference	Reference	Reference	Reference
41–48 h	0.88 (0.81–0.97)	0.95 (0.82–1.10)	0.97 (0.88–1.06)	0.91 (0.82–1.01)	1.02 (0.80–1.30)	0.96 (0.86–1.06)
49–54 h	0.87 (0.78–0.96)	0.82 (0.70–0.97)	0.90 (0.81–1.00)	1.04 (0.90–1.19)	1.12 (0.84–1.49)	0.87 (0.76–1.00)
≥55 h	0.84 (0.77–0.92)	0.82 (0.72–0.94)	0.83 (0.76–0.92)	0.79 (0.70–0.89)	0.88 (0.70–1.11)	0.80 (0.71–0.90)
**Age**						
19–29	Reference	Reference	Reference	Reference	Reference	Reference
30–39	1.07 (0.93–1.23)	0.94 (0.76–1.15)	1.42 (1.24–1.64)	1.09 (0.96–1.24)	1.69 (1.21–2.34)	1.58 (1.40–1.79)
40–49	1.32 (1.14–1.52)	0.87 (0.71–1.07)	1.36 (1.17–1.58)	1.39 (1.23–1.57)	1.91 (1.41–2.59)	1.46 (1.29–1.65)
50–59	1.50 (1.30–1.74)	0.79 (0.64–0.97)	1.26 (1.08–1.47)	1.55 (1.36–1.77)	1.85 (1.37–2.50)	1.64 (1.43–1.89)
≥60	1.73 (1.47–2.04)	0.66 (0.53–0.81)	1.33 (1.12–1.57)	1.54 (1.31–1.80)	1.39 (1.02–1.90)	1.29 (1.10–1.52)
**Education level**						
Middle school or below	Reference	Reference	Reference	Reference	Reference	Reference
High school	1.52 (1.37–1.69)	1.61 (1.43–1.80)	2.13 (1.89–2.40)	1.52 (1.35–1.70)	2.05 (1.66–2.52)	2.19 (1.94–2.46)
College or above	1.98 (1.76–2.23)	1.74 (1.50–2.02)	2.97 (2.61–3.38)	2.14 (1.87–2.44)	3.33 (2.47–4.51)	3.64 (3.17–4.19)
**Marital status**						
Married	Reference	Reference	Reference	Reference	Reference	Reference
Unmarried/others	0.80 (0.73–0.88)	0.66 (0.58–0.75)	0.91 (0.82–1.01)	0.98 (0.90–1.06)	0.90 (0.77–1.06)	0.96 (0.88–1.04)
**Income level**						
Q1	Reference	Reference	Reference	Reference	Reference	Reference
Q2	1.03 (0.88–1.19)	1.24 (1.08–1.43)	1.42 (1.21–1.66)	1.23 (1.07–1.41)	1.38 (1.16–1.65)	1.31 (1.14–1.50)
Q3	1.26 (1.09–1.47)	1.55 (1.34–1.80)	1.60 (1.36–1.88)	1.40 (1.22–1.61)	1.60 (1.31–1.95)	1.50 (1.31–1.72)
Q4	1.64 (1.41–1.91)	1.89 (1.62–2.20)	1.64 (1.40–1.92)	1.61 (1.39–1.85)	2.10 (1.67–2.63)	1.62 (1.41–1.87)
**Occupation**						
White collar	Reference	Reference	Reference	Reference	Reference	Reference
Service/sales worker	0.85 (0.77–0.95)	0.85 (0.73–1.00)	1.02 (0.91–1.13)	0.91 (0.83–1.00)	0.85 (0.65–1.11)	0.98 (0.89–1.09)
Blue collar	0.93 (0.85–1.01)	0.55 (0.48–0.63)	0.82 (0.76–0.90)	0.74 (0.66–0.83)	0.54 (0.41–0.71)	0.74 (0.66–0.83)
**Employment type**						
Permanent	Reference	Reference	Reference	Reference	Reference	Reference
Fixed-term	0.63 (0.57–0.71)	0.84 (0.72–0.97)	0.95 (0.84–1.07)	0.86 (0.78–0.94)	0.77 (0.63–0.95)	0.93 (0.85–1.03)
Self-employment	0.63 (0.58–0.68)	0.64 (0.57–0.71)	0.94 (0.87–1.02)	0.72 (0.66–0.79)	0.49 (0.40–0.59)	0.91 (0.83–1.00)
**Shift work**						
No	Reference	Reference	Reference	Reference	Reference	Reference
Yes	1.11 (1.03–1.19)	0.97 (0.88–1.07)	0.74 (0.69–0.80)	0.95 (0.88–1.03)	0.98 (0.85–1.14)	0.73 (0.68–0.79)

OR, odds ratio; CI, confidence interval

**Fig. 2:**
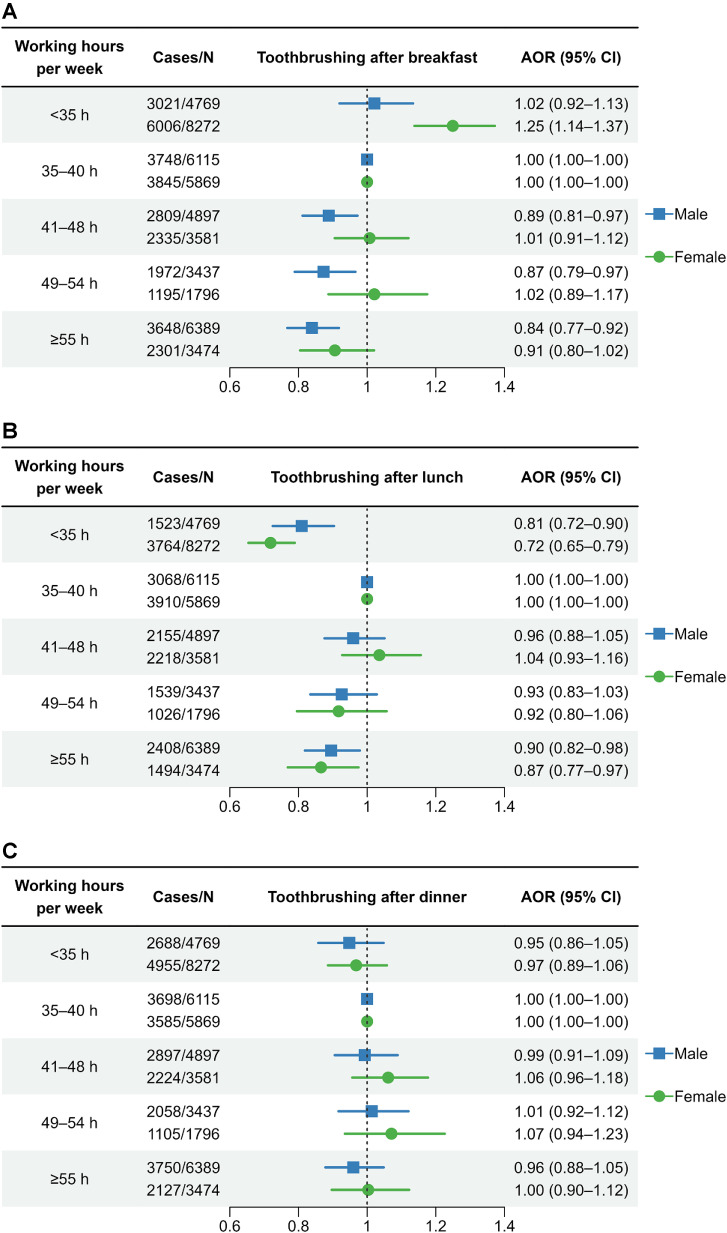
Association between working hours and post-meal toothbrushing (AOR, adjusted odds ratio; CI, confidence interval)

Figure 2 shows the association between working hours and post-mealtime toothbrushing. In male workers, the adjusted OR (95% CI) of the association between working ≥55 h/week, compared with 35–40 h/week, each post-mealtime toothbrushing was 0.84 (0.77–0.92) for breakfast, 0.90 (0.82–0.98) for lunch, 0.96 (0.88–1.05) for dinner. In female workers, the adjusted OR (95% CI) of the association between working ≥55 h/week, compared with 35–40 h/week, each post-mealtime toothbrushing was 0.91 (0.80–1.02) for breakfast, 0.87 (0.77–0.97) for lunch, and 1.00 (0.90–1.12) for dinner.

## 4. Discussion

Our study demonstrated that individuals working long hours were less likely to participate in dental check-ups and use interdental cleaning devices. Additionally, working >48 h/week is associated with non-adherence to the recommended daily toothbrushing frequency in male workers. Consequently, our findings suggest that policy efforts are required to closely monitor oral health outcomes and promote preventive oral health behaviors among those working long hours and overtime.

As shown in our descriptive analysis, approximately 34% of workers participated in annual dental check-ups, a figure similar to that reported among Japanese workers [13]. Additionally, the proportion of workers exposed to long working hours (≥55 h/week, 25.3%) was higher than in others regions, such as Europe [1]. These findings suggest the need for policy efforts to promote preventive oral health behaviors among workers and reduce long working hours.

Our findings are consistent with those of previous studies, showing that those working long hours are less likely to participate in preventive dental visits or receive dental care for symptom management. For instance, frequent overtime was negatively associated with preventive dental visits among Japanese workers [13] and working >60 h/week was negatively associated with attendance at dental check-ups among workers in the United States [12]. Additionally, previous studies have consistently shown that those working long hours are more likely to have untreated dental needs [7, 31]. This study adds to the existing body of literature by elaborating that not only the use of dental health services but also daily oral health behaviors, are affected by long working hours.

One potential mechanism through which long working hours may contribute to unfavorable oral health behaviors is the time pressure they place on workers and the challenge they pose to engaging in oral healthcare. This hypothesis is supported by previous studies showing that long working hours act as a time barrier for workers to utilize healthcare services [7, 12, 13] and other well-known health-promoting behaviors such as leisure-time physical activity [32]. In particular, this would explain our observation that long working hours are associated with the omission of tooth brushing during the morning and lunch hours; these are periods during which individuals with long working hours are more likely to face time constraints posed by commuting or daily work commitments.

One unexpected finding was that short working hours (<35 h/week) were also associated with reduced tooth brushing frequency and omission of tooth brushing after lunch. Our results are consistent with those of a previous study showing that part-time workers are less likely to brush their teeth than full-time workers [19]. Firstly, part-time workers may be less likely to be guaranteed lunch breaks. Secondly, part-time workers may have limited access to sanitary facilities in which to practice oral hygiene. These findings underscore the need for policy efforts aimed at promoting oral health behaviors among part-time workers.

From a gender perspective, the overall prevalence of preventive oral health behaviors was higher among female workers, which is in line with previous studies [20, 29]. This can be attributed to the fact that women are more likely to view oral health as having a more significant influence on their overall appearance and well-being [33] and have better oral health literacy than men [34]. While our findings suggest that long working hours are associated with poor oral health behaviors in both men and women, long working hours are negatively associated with toothbrushing twice a day in men, but not in women. In the context of societal norms, female workers are often expected to prioritize morning hygiene routines more strictly than men [35], which may result in continued tooth brushing despite time constraints in the morning.

Our findings have important public health implications. Our study suggests that poor oral health behaviors may be a crucial factor linking long working hours to adverse dental conditions. Therefore, active policy interventions, aimed at improving the oral health behaviors of workers exposed to long working hours, are required [36, 37]. For instance, considering that these workers have limited access to dental visits owing to time constraints, dental check-ups at the workplace could aid in monitoring and managing their dental condition [38]. Previous research has also documented that workplace interventions, aimed at enhancing oral health behaviors, can lead to improvements in the quality of self-care practices and dental knowledge, attitudes, and behaviors [39]. Additionally, our analysis shows that not only working hours but also low education levels, blue-collar occupations, and low income levels are associated with less engagement in preventive oral health behaviors. Therefore, this suggests the need for proactive policy efforts to promote oral health behaviors among workers with low socio-economic status.

Our study had several limitations. Firstly, because of the cross-sectional design, we could not establish the true causal effect of long working hours on preventive oral health behaviors. Therefore, future longitudinal studies are required to clarify the temporal relationship between long working hours and preventive oral health behaviors. Secondly, there is a possibility of unmeasured confounding factors, such as previous dental history and oral health literacy, introducing bias into our estimations. Additionally, we could not consider whether the workers were employed part-time or full-time owing to the lack of information. Thirdly, all variables were self-reported by the survey participants, which may have introduced measurement errors such as recall bias. Finally, in addition to tooth brushing frequency, the technique and duration of tooth brushing are important factors in oral hygiene. Unfortunately, owing to a lack of information, we could not account for these behavioral factors. Therefore, future research could derive valuable insights by incorporating survey questionnaires covering a broader range of information related to preventive oral health behaviors.

## 5. Conclusion

In this study, we showed that long working hours were associated with a decrease in several preventive oral health behaviors such as regular dental check-ups, adherence to the recommended daily toothbrushing frequency, and the use of interdental cleaning devices. Moreover, socio-economic status, including educational attainment, income level, and blue-collar occupations, are major determinants of preventive oral health behaviors among Korean workers. Policy interventions are required to monitor and promote favorable oral health behaviors within working populations.

## Supplementary information

**Additional file 1:** **Table S1** Characteristics of the study sample according to working hours per week. **Table S2** Association between working hours and each preventive oral health behavior according to occupational groups and employment types. **Figure S1** Weight-adjusted prevalence of preventive oral health behaviors among workers.

## Declarations

### Availability of data and material

The KNHANES dataset is available online at https://knhanes.kdca.go.kr.
