# Supplementary material for: Long working hours and preventive oral health behaviors: a nationwide study in Korea (2007–2021)
Source: Environ Health Prev Med. 2024 Sep 12;29:48. doi: 10.1265/ehpm.24-00102 (PMC11413638; doi:10.1265/ehpm.24-00102)
Supplement: Supplementary file 1 — Additional file 1: Table S1 Characteristics of the study sample according to working hours per week. Table S2 Association between working hours and each preventive oral health behavior according to occupational groups and employment types. Figure S1 Weight-adjusted prevalence of preventive oral health behaviors among workers. [file ehpm-29-048-s001.docx]

**Table S1** Characteristics of the study sample according to working hours per week.

|  | **Male** | | | | | | **Female** | | | | | |
| --- | --- | --- | --- | --- | --- | --- | --- | --- | --- | --- | --- | --- |
|  | **Total** | **Working hours per week** | | | | | **Total** | **Working hours per week** | | | | |
|  |  | **<35 h** | **35–40 h** | **41–48 h** | **49–54 h** | **≥55 h** |  | **<35 h** | **35–40 h** | **41–48 h** | **49–54 h** | **≥55 h** |
|  | **N (%)** | **N (%)** | **N (%)** | **N (%)** | **N (%)** | **N (%)** | **N (%)** | **N (%)** | **N (%)** | **N (%)** | **N (%)** | **N (%)** |
| **Age** |  |  |  |  |  |  |  |  |  |  |  |  |
| 19–29 | 2543 (14.7) | 677 (23.6) | 575 (13.2) | 458 (13.1) | 351 (14.6) | 482 (11.8) | 3207 (19.9) | 1015 (18.2) | 976 (22.1) | 628 (24.3) | 317 (25.1) | 271 (11.9) |
| 30–39 | 5383 (24.1) | 341 (9.8) | 1422 (26.0) | 1251 (28.2) | 941 (29.3) | 1428 (25.2) | 4061 (18.5) | 1219 (16.0) | 1353 (22.7) | 788 (22.2) | 328 (19.0) | 373 (12.4) |
| 40–49 | 6098 (26.3) | 493 (13.7) | 1748 (29.9) | 1346 (29.3) | 961 (28.3) | 1550 (27.4) | 5335 (24.9) | 1667 (22.6) | 1613 (28.0) | 867 (24.7) | 412 (23.7) | 776 (25.5) |
| 50–59 | 5577 (21.4) | 941 (22.1) | 1391 (21.7) | 1051 (20.2) | 696 (19.7) | 1498 (22.8) | 5250 (21.5) | 1678 (20.2) | 1225 (19.3) | 751 (18.7) | 421 (21.2) | 1175 (32.2) |
| ≥60 | 6006 (13.4) | 2317 (30.9) | 979  (9.2) | 791 (9.2) | 488 (8.1) | 1431 (12.8) | 5139 (15.2) | 2693 (23.0) | 702  (7.8) | 547 (10.2) | 318 (10.9) | 879 (17.9) |
| **Education level** |  |  |  |  |  |  |  |  |  |  |  |  |
| Middle school or  below | 5814 (16.7) | 1767 (27.6) | 809  (9.7) | 858 (12.8) | 591 (13.5) | 1789 (21.5) | 7559 (25.8) | 3196 (30.0) | 1117 (14.7) | 913 (20.5) | 582 (25.3) | 1751 (42.7) |
| High school | 8945 (37.7) | 1756 (43.2) | 1859 (32.0) | 1694 (36.5) | 1178 (36.3) | 2458 (41.8) | 7492 (35.8) | 2590 (36.4) | 1988 (35.4) | 1185 (34.5) | 585 (34.9) | 1144 (37.1) |
| College or above | 10,848 (45.6) | 1246 (29.2) | 3447 (58.4) | 2345 (50.8) | 1668 (50.2) | 2142 (36.7) | 7941 (38.4) | 2486 (33.7) | 2764 (49.9) | 1483 (45.0) | 629 (39.8) | 579 (20.2) |
| **Marital status** |  |  |  |  |  |  |  |  |  |  |  |  |
| Married | 20,350 (74.1) | 3546 (64.8) | 4870 (74.5) | 3898 (75.3) | 2738 (74.8) | 5298 (78.3) | 15,491 (64.3) | 5493 (64.7) | 3955 (63.7) | 2337 (61.1) | 1175 (59.7) | 2531 (70.3) |
| Unmarried/others | 5257 (25.9) | 1223 (35.2) | 1245 (25.5) | 999 (24.7) | 699 (25.2) | 1091 (21.7) | 7501 (35.7) | 2779 (35.3) | 1914 (36.3) | 1244 (38.9) | 621 (40.3) | 943 (29.7) |
| **Income level** |  |  |  |  |  |  |  |  |  |  |  |  |
| Q1 | 2625  (7.7) | 1168 (19.6) | 368  (4.9) | 326 (5.1) | 189 (4.0) | 574  (7.1) | 3426 (12.1) | 1895 (18.7) | 469  (7.0) | 336 (7.9) | 188 (7.7) | 538 (12.7) |
| Q2 | 6143 (23.4) | 1342 (27.5) | 1125 (18.3) | 1118 (22.2) | 795 (22.6) | 1763 (27.2) | 5598 (23.8) | 2127 (25.9) | 1221 (20.2) | 808 (21.7) | 432 (23.7) | 1010 (28.2) |
| Q3 | 7976 (32.8) | 1115 (25.9) | 1968 (33.0) | 1704 (36.4) | 1141 (34.4) | 2048 (33.2) | 6559 (30.1) | 2115 (27.6) | 1806 (31.9) | 1098 (31.8) | 541 (31.6) | 999 (30.2) |
| Q4 | 8863 (36.1) | 1144 (27.0) | 2654 (43.8) | 1749 (36.3) | 1312 (39.0) | 2004 (32.5) | 7409 (33.9) | 2135 (27.8) | 2373 (40.9) | 1339 (38.5) | 635 (36.9) | 927 (28.8) |
| **Occupation** |  |  |  |  |  |  |  |  |  |  |  |  |
| White collar | 9493 (39.6) | 1250 (30.0) | 3266 (55.1) | 2028 (43.5) | 1416 (42.4) | 1533 (25.8) | 8650 (41.7) | 2597 (34.9) | 3194 (57.3) | 1692 (50.8) | 676 (43.1) | 491 (17.9) |
| Service/sales worker | 3800 (16.6) | 666 (19.0) | 667 (11.7) | 612 (13.6) | 452 (14.5) | 1403 (23.4) | 6475 (29.4) | 2260 (30.4) | 1194 (20.6) | 865 (24.6) | 517 (28.6) | 1639 (49.4) |
| Blue collar | 12,314 (43.8) | 2853 (51.0) | 2182 (33.2) | 2257 (42.8) | 1569 (43.1) | 3453 (50.8) | 7867 (28.9) | 3415 (34.7) | 1481 (22.1) | 1024 (24.6) | 603 (28.3) | 1344 (32.7) |
| **Employment type** |  |  |  |  |  |  |  |  |  |  |  |  |
| Permanent | 12,642 (53.1) | 813 (18.8) | 4048 (68.6) | 2960 (63.4) | 2121 (65.1) | 2700 (44.7) | 9898 (46.3) | 2089 (26.2) | 3941 (69.5) | 2127 (62.4) | 922 (56.9) | 819 (27.8) |
| Fixed-term | 3453 (14.3) | 1495 (35.6) | 645 (10.9) | 465 (10.4) | 298 (9.4) | 550 (10.0) | 5798 (25.9) | 3576 (45.1) | 961 (16.4) | 522 (15.4) | 251 (14.2) | 488 (14.9) |
| Self-employment | 9512 (32.6) | 2461 (45.6) | 1422 (20.5) | 1472 (26.2) | 1018 (25.6) | 3139 (45.3) | 7296 (27.8) | 2607 (28.7) | 967 (14.0) | 932 (22.2) | 623 (28.9) | 2167 (57.3) |
| **Shift work** |  |  |  |  |  |  |  |  |  |  |  |  |
| No | 17,544 (68.6) | 3391 (67.8) | 4581 (75.4) | 3575 (73.4) | 2350 (68.2) | 3647 (58.8) | 15,989 (70.3) | 5690 (67.3) | 4427 (76.1) | 2549 (73.1) | 1246 (71.7) | 2077 (62.6) |
| Yes | 8063 (31.4) | 1378 (32.2) | 1534 (24.6) | 1322 (26.6) | 1087 (31.8) | 2742 (41.2) | 7003 (29.7) | 2582 (32.7) | 1442 (23.9) | 1032 (26.9) | 550 (28.3) | 1397 (37.4) |

Survey weights were adjusted.
The exact values for income level categorizations are shown in the analysis guideline of the KNHANES (28).

**Table S2** Association between working hours and each preventive oral health behavior according to occupational groups and employment types.

|  | **Occupational groups** | | | **Employment type** | | |
| --- | --- | --- | --- | --- | --- | --- |
|  | **White collar** | **Service or sales** | **Blue collar** | **Regular** | **Fixed-term** | **Daily** |
|  | **Adjusted model** | **Adjusted model** | **Adjusted model** | **Adjusted model** | **Adjusted model** | **Adjusted model** |
|  | **OR (95% CI)** | **OR (95% CI)** | **OR (95% CI)** | **OR (95% CI)** | **OR (95% CI)** | **OR (95% CI)** |
| **Participation in dental check-up** |  |  |  |  |  |  |
| Working hours per week |  |  |  |  |  |  |
| <35 h | 1.01 (0.91–1.12) | 1.04 (0.89–1.22) | 0.99 (0.88–1.12) | 0.93 (0.84–1.04) | 1.01 (0.86–1.17) | 1.15 (1.01–1.31) |
| 35–40 h | Reference | Reference | Reference | Reference | Reference | Reference |
| 41–48 h | 0.89 (0.81–0.98) | 0.83 (0.70–0.98) | 0.93 (0.82–1.06) | 0.89 (0.81–0.97) | 0.91 (0.74–1.12) | 0.95 (0.81–1.11) |
| 49–54 h | 0.96 (0.85–1.07) | 0.95 (0.77–1.17) | 0.84 (0.73–0.97) | 0.91 (0.82–1.01) | 1.04 (0.79–1.37) | 0.89 (0.76–1.06) |
| ≥55 h | 0.83 (0.73–0.93) | 0.78 (0.66–0.92) | 0.85 (0.76–0.96) | 0.78 (0.71–0.87) | 0.99 (0.79–1.23) | 0.90 (0.78–1.03) |
| **Toothbrushing ≥twice a day** |  |  |  |  |  |  |
| Working hours per week |  |  |  |  |  |  |
| <35 h | 0.82 (0.63–1.05) | 0.94 (0.70–1.25) | 0.91 (0.78–1.05) | 0.76 (0.60–0.96) | 0.99 (0.79–1.24) | 0.83 (0.70–0.99) |
| 35–40 h | Reference | Reference | Reference | Reference | Reference | Reference |
| 41–48 h | 0.91 (0.72–1.16) | 1.02 (0.74–1.43) | 0.99 (0.83–1.17) | 0.98 (0.81–1.19) | 0.98 (0.73–1.33) | 0.95 (0.77–1.18) |
| 49–54 h | 0.69 (0.53–0.90) | 0.98 (0.69–1.39) | 0.94 (0.77–1.14) | 0.88 (0.71–1.08) | 0.76 (0.53–1.08) | 0.93 (0.74–1.17) |
| ≥55 h | 0.64 (0.50–0.83) | 0.85 (0.64–1.12) | 0.92 (0.79–1.07) | 0.81 (0.66–0.99) | 0.89 (0.67–1.19) | 0.85 (0.70–1.02) |
| **Use of interdental cleaning device** |  |  |  |  |  |  |
| Working hours per week |  |  |  |  |  |  |
| <35 h | 1.16 (1.05–1.29) | 1.13 (0.97–1.31) | 1.07 (0.94–1.22) | 1.10 (0.98–1.22) | 1.15 (0.99–1.34) | 1.18 (1.03–1.36) |
| 35–40 h | Reference | Reference | Reference | Reference | Reference | Reference |
| 41–48 h | 0.95 (0.87–1.05) | 0.96 (0.81–1.14) | 0.97 (0.85–1.11) | 0.95 (0.88–1.04) | 0.91 (0.73–1.13) | 1.08 (0.93–1.27) |
| 49–54 h | 0.87 (0.77–0.98) | 0.83 (0.68–1.01) | 0.93 (0.80–1.09) | 0.84 (0.76–0.93) | 0.95 (0.72–1.24) | 1.06 (0.88–1.26) |
| ≥55 h | 0.80 (0.70–0.91) | 0.79 (0.68–0.92) | 0.86 (0.75–0.98) | 0.80 (0.71–0.88) | 0.77 (0.61–0.96) | 0.94 (0.82–1.08) |

OR, odds ratio; CI, confidence interval

**
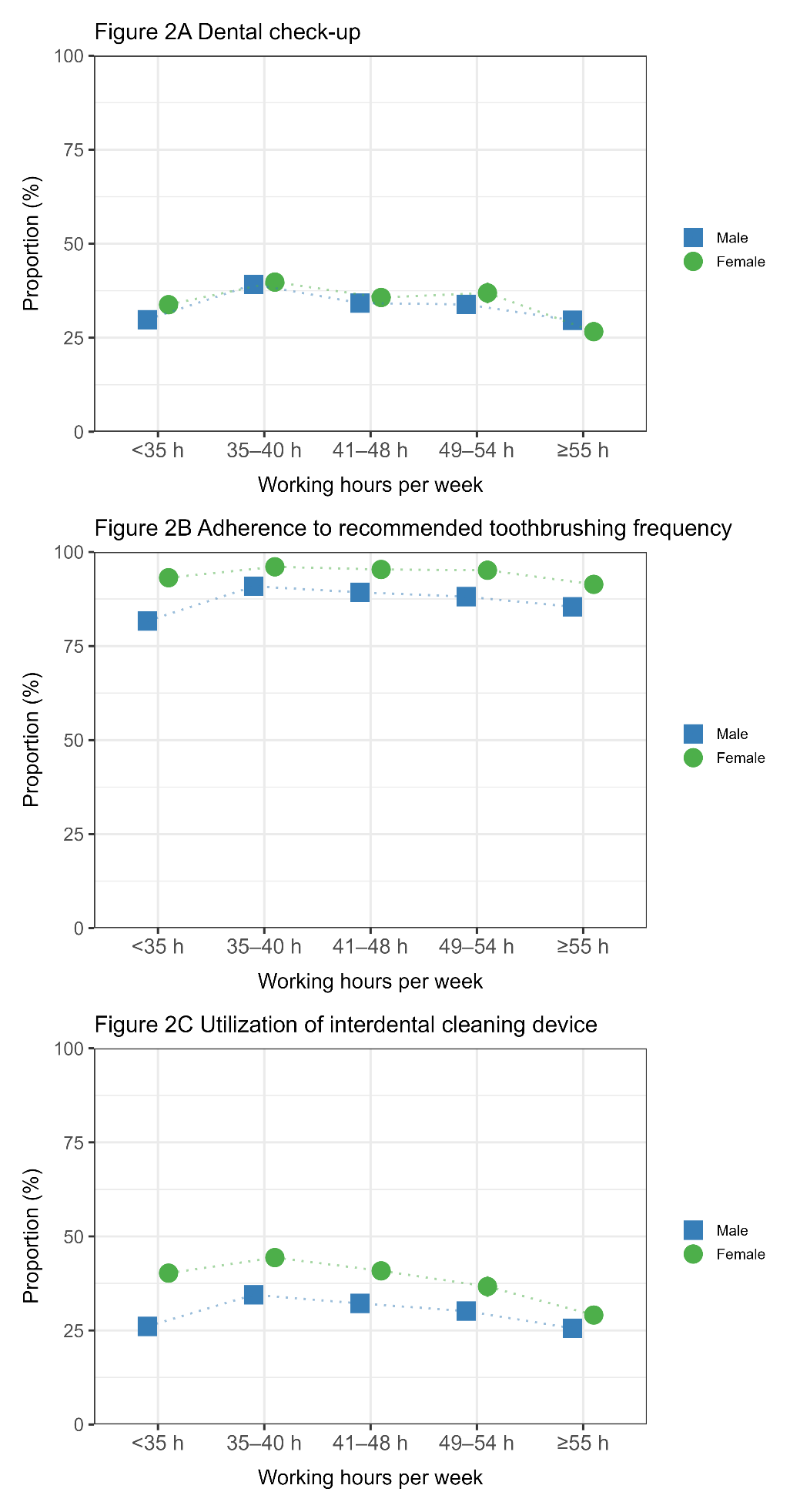
**

**Figure S1** Weight-adjusted prevalence of preventive oral health behaviors among workers
